# JEasyTFM: an open-source software package for the analysis of large 2D TFM data within ImageJ

**DOI:** 10.1093/bioadv/vbad156

**Published:** 2023-10-26

**Authors:** Philippe Carl, Philippe Rondé

**Affiliations:** Université de Strasbourg, CNRS, LBP UMR 7021, F-67401 Illkirch Cedex, France; Université de Strasbourg, CNRS, LBP UMR 7021, F-67401 Illkirch Cedex, France

## Abstract

**Motivation:**

Cells adhering to the extracellular matrix can sense and respond to a wide variety of chemical and physical features of the adhesive surface. Traction force microscopy (TFM) allows determining the tensile forces exerted by the cells on their substrate with high resolution.

**Results:**

To allow broad access of this techniques to cell biology laboratories we developed JeasyTFM, an open-source ImageJ package able to process multi-color and multi-position time-lapse pictures thus suitable for the automatic analysis of large TFM data.

**Availability and implementation:**

JEasyTFM is implemented as an ImageJ plugin and available at: http://questpharma.u-strasbg.fr/JEasyTFM.html.

## 1 Introduction

Force is widely used in biological systems to influence cellular programs like cell migration, differentiation, and growth. Indeed, force sensing by cells is critical for their proper function, but how cells transform a force stimulus into a chemical response remains unclear. To decipher the fundamental mechanisms of force-induced control of biochemical pathways, more systematic studies of mechanotransduction in living cells are needed. To do so, traction force microscopy (TFM) (Gil-Redondo *et al.* 2023, Issler *et al.* 2023, Rajendran *et al.* 2023) has emerged as a method of choice to produce spatially resolved measurement of the traction exerted on the substrate by a single motile cell. TFM relates to the elastic substrate method which extracts force calculation from displacement data of fluorescent beads embedded into the elastic substrate.

Several algorithms for processing 2D TFM data have already been published, but widely based upon Matlab^®^ implementation (Sabass *et al.* 2008, Schwarz and Soine 2015) or Abaqus™ (Condor and Garcia-Aznar 2019). These software are however not freely available, thus strongly limiting the adoption of such technique. In parallel, the freely available and widely used open source software ImageJ (Schneider *et al.* 2012) (http://imagej.net/ij/) has become the standard tool for the analysis of microscopy pictures. Up today, there are already some freely available codes build upon ImageJ plugins (Martiel *et al.* 2015) describing processes allowing the calculation of the force maps by means of several manual analysis steps similarly to another freely available code developed under Python (Bauer *et al.* 2021). Nevertheless, this limits the use of TFM to experts reasonably familiar with using several plugins in a row and makes the analysis of time-lapse experiments and/or on large amount of cells extremely tedious and strongly time consuming. These limitations explain why, although TFM becomes quite a standard technique to analyze mechanotransduction only a few publications focused on kinetic studies (Rieu *et al.* 2009, Plotnikov *et al.* 2012, Morin *et al.* 2014). However kinetic analysis is essential to understand how cells integrate forces to promote timely correlated biochemical response leading to different cell fate decision. Thus, building up on some already freely existing tools (Schwarz *et al.* 2002, Martiel *et al.* 2015), we created an ImageJ package, available as open-source and capable of a fully automated processing and analysis of multi-color beads, multi-position, and multi-channel time-lapse TFM data.

## 2 Methods

Primary FAK−/− mouse embryonic fibroblasts (MEFs) were maintained in DMEM supplemented with 10% FBS, 100 U/ml penicillin, and 100 mg/ml streptomycin as previously described (Deramaudt *et al.* 2011). Thirty hours before cell recording, 2.10^5^ primary FAK−/− cells were seeded into a 6-well plate and transfected with FAK-GFP using Lipofectamine 2000 (Invitrogen) according to the manufacturer’s directions. 10^5^ cells were then plated for 6 h on a previously prepared poly-acrylamide gel substrate containing fluorescent beads (Fluospheres Dark Red, F8807, Thermo Fisher) as previously described (Yeung *et al.* 2005).

Image acquisitions were performed using an iMIC microscope (Till Photonics, Munich, Germany) equipped with a Spectra X (Lumencor) LED light, an Olympus (Rungis, France) 60× TIRFM (1.45 NA) objective, an automated stage (Prior, Cambridge, UK) and a piezo z-controller. During acquisition, cells were maintained at 37°C in a 5% CO_2_ humidified atmosphere using an environmental control system (Life Imaging Services). Images were acquired every 5 min on an ORCA-Flash4.0 V2 CMOS camera (Hamamatsu Photonics, Tsukuba, Japan).

The microscopy experiments consisted first on identifying the x–y stage positions corresponding to 20–30 isolated cells in order to be able to measure their traction forces without being constrained by measurements errors introduced by force contribution of neighboring cells. Once the x–y stage positions for each chosen cell were recorded, each reference z positions based upon the position of the top beads within the gel being perfectly in focus were carefully defined. A single time point two channels acquisition (i.e. Bright Field and GFP EPI) of the cells together with a single time point one channel acquisition corresponding to the embedded beads were then recorded for each previously defined x–y positions. As it is important to acquire the best focused image for accurate tracking of the beads displacement, for each x–y positions a z-stack (14 images each 200 nm steps depth) is also recorded. Time-lapse recording at a frequency of one set of images every 5 min for 4 h was then achieved followed by incubation of the cells with PBS/Trypsin (Sabass *et al.* 2008, Martiel *et al.* 2015, Schwarz and Soine 2015, Condor and Garcia-Aznar 2019, Bauer *et al.* 2021) to detach the cells from the substrate. Then, for each x–y positions a z-stack with the channel corresponding to the embedded beads was acquired to record the beads positions at equilibrium. Of note for each time point and each position, 28 images are recorded for a total of 1344 images for one cell thus, depending of the cells sensitivity to photo-damage, to the precision of the x–y stage and piezo, or to the correlation between cell behavior/protein dynamic and traction force to be achieved, the frequency of imaging, the depth of the z-stack or the number of channels being recorded could be reduced. On a typical desktop computer (CPU I7-12700 3.7 GHz, 16 GB memory) under Windows 10 running ImageJ 1.54c27 (Java 1.8.0_345–64Bits) processing of the force calculation applied on a 4-h time-lapse of 29 chosen cells, two channels (Bright field and beads) with 14 acquired z-slices at a frequency of one set of images every 5 min took 19 h and 40 min (i.e. about 40 min for analyzing the data for one cell) to be performed up to the Traction_Force>Force_Superposition step.

## 3 Results

**Figure 1.**
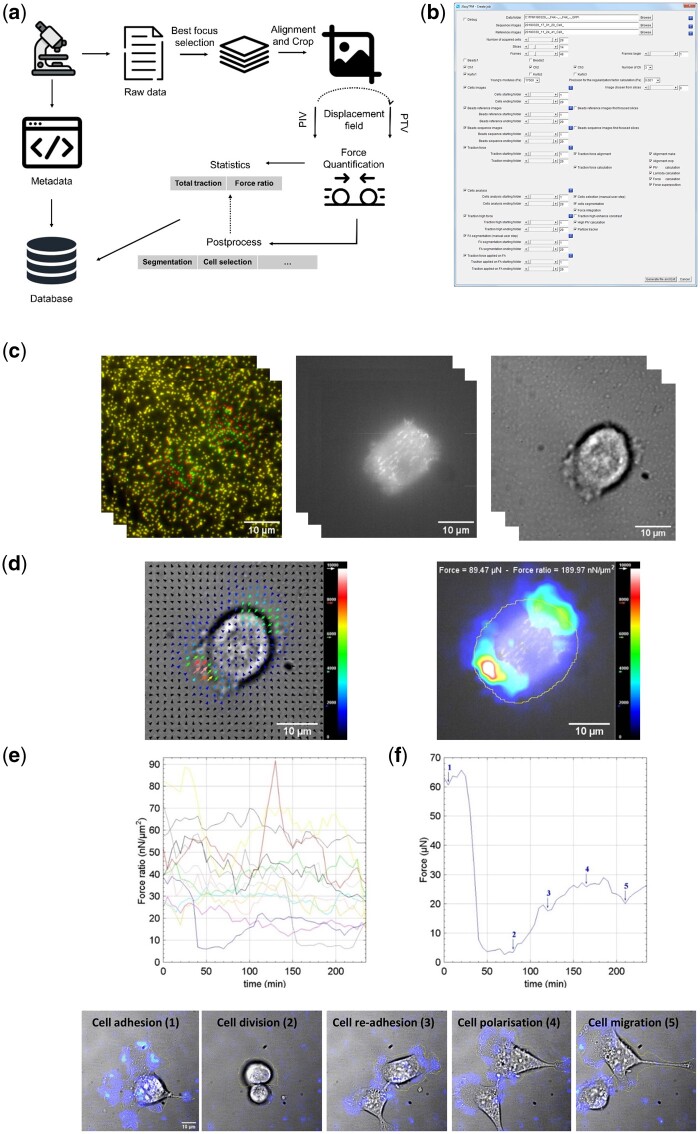
Overview of JEasyTFM functionality, workflow, and analysis. (a) JEasyTFM functionality: Metadata, video, statistics, and metrics derived from pre and postprocessing algorithms are added to the database after processing. (b) JEasyTFM Graphical User Interface displaying TFM data. (c) The software loads and processes raw video files. Processing includes best focused picture selection of all time-series and z-series in all selected channels (beads, left; cell fluorescence, middle; cell bright field, right) in both constrained and equilibrium conditions. Beads displacements are shown (left) by superposition of beads images before (green) and after cell-substratum adhesive contacts disruption (red). Drift correction and slice alignment algorithms allow computation of the displacement of each bead relative to its undisturbed position. (d) (left) Traction forces are calculated from the displacement field using the Fourier Transform Traction Cytometry (FTTC) method and traction vectors are displayed superimposed on the fluorescence picture of the cells. (right) Automated cell selection for total force and total force ratio calculation. (e) Force–time traces of individual cells in each movie are calculated and stored in an interactive database for export to standard data sheets. (f) Video TFM analysis of single cell allows correlation of total force with cell behavior properties.

JEasyTFM uses ImageJ environment and is therefore compatible with the majority of data output formats thanks to the BioFormats plug-in. JEasyTFM integrates and automates common procedures in TFM data analysis such as best focused picture selection, drift correction, slice alignment, displacement field computation, traction force calculation, cell segmentation, and force integration. This pipeline is summarized in Fig. 1a. The software provides tools for interacting visually with the data. From the user interface, the package is split into two subunits. A first unit displays a Graphical User Interface window to create the analysis job to be performed and saves it as an ASCII file upon validation (Fig. 1b). The second unit reads this file and launches sequentially the defined analysis steps. This procedure allows safeguarding the used analysis but also, in the case of shared resources, to process the analysis job on defined times. On top of this feature, a copy of the job file is made upon launching the analysis. This job file is updated and saved each time an analysis step is completed, thereby insuring full traceability and the possibility of splitting the analysis into several steps. The input pictures files must be in OME TIF format with the z-stack followed by the time series pictures within a single file for each x-y position and channel. As results of the analysis, at each analysis steps (Fig. 1b), several files in TIF, JPEG, or ASCII formats are created and saved in different folders which make it possible to follow the analysis procedures in detail and easily locate an eventual issue. ASCII files can be further imported into spreadsheets or statistical software (Fig. 1e).

### 3.1 JeasyTFM processing and application

To demonstrate the capabilities of JEasyTFM, we process multi-position two channels time-lapse experiments to follow more than 60 cells during 4 h at 3.33×10^−3^ Hz. TFM microscopy were performed on primary FAK−/− MEFs plated on poly-acrylamide gel substrate containing fluorescent beads. Images were acquired using a time-lapse microscope equipped with an automated stage and a piezo z-controller at 37°C in a 5% CO_2_ humidified atmosphere.

#### 3.1.1 4D images best focus determination and alignment

For each position and time-point, a z-stack is acquired in both the bright field and fluorescent (i.e. beads) channel. The software allows automatic selection and repositioning of the best focused images in all channels and time-points (Fig. 1). For each acquired channel, the selection is made using either a Kurtosis (Pearson 1905) or EDF (Forster *et al.* 2004) algorithm. We found that the Kurtosis algorithm is better suited for the Bright Field picture whereas the EDF algorithm is highly efficient for selecting fluorescent images. One of the critical points during TFM processing is the correct alignment of each cell/beads pair images acquired during the time series and at equilibrium. For example, at the end of the experiment, force-removing compounds are added to the sample in order to obtain a reference image, which may cause substantial shift in the focal plane. Thus, once all the best focused pictures of the time lapse have been processed, the software allows calculation of the maximum displacement during image alignment through all the time-lapse beads pictures and cropped them out subsequently. Following translation and cropping, corrections that have been applied to the beads pictures are similarly applied to the cells pictures through the time-lapse data. This allows perfect focusing and repositioning of each image of the dataset.

#### 3.1.2 Calculation of the bead displacement field

Measuring changes in traction due to applied forces requires images during and after the force application. Beads displacements are calculated from the beads images taken sequentially and the images at equilibrium. Images of beads before and after force-induced movement are converted to displacement data, also called deformation field, by either interpolating bead movements using particle image velocimetry (PIV) or direct tracking of the beads using particle tracking velocimetry (PTV). As reported previously, the PIV method splits the beads images into large number of interrogation windows thus allowing to calculate a displacement vector for each window (Adrian 1991, 2005). However, limitations of the PIV method such as inherent averaging effects not appropriate for high-resolution TFM have been identified. Thus, to overcome these limitations, JEasyTFM provide a particle tracking (PTV) algorithm founded upon the ParticleTracker_2D (Sbalzarini and Koumoutsakos 2005). This code is based on a scoring between the differences of the beads positions, gray values, and derivations of the gray values upon the definition of an exploration radius. However, this code displays also limitations as it does not always allow a correct tracking of the beads over the whole picture. Indeed, beads couples displaying large displacements are missed if the exploration radius is set to a value that is smaller than the biggest bead displacement within the picture. Thus, to improve the quality of the beads tracking routine we modified the original ParticleTracker_2D algorithm by implementing the results of the displacements maps obtained by the PIV algorithm as starting positions for the tracking algorithm. This allows to use a very small exploration radius suitable for high-resolution TFM provided by computing two colors nanobeads displacement (Supplementary Fig. S1).

#### 3.1.3 Force quantification and representation

Traction stresses are estimated from the displacement data using the Fourier Transform Traction Cytometry (FTTC) method as reported previously (Martiel *et al.* 2015, Schwarz and Soine 2015, Bauer *et al.* 2021). Briefly the linear elasticity theory, relates the stress field ***F***(***r***) and displacement field ***u***(***r***) where ***r***(*x*, *y*) represent a point at the surface of the gel, by the following equation:


(1) $$u\left(r\right)=\int G(r-r^{\prime })F(r^{\prime })dr'$$

with ***G***(***r*−*r′***) the Green function that depend on the boundary conditions and gel properties.

By assuming that the displacement vectors are 2D in the *x*–*y* plane and given that our substrate with Young’s modulus equal to *E* is mostly composed of water and thus has a Poisson ratio ν close to 0.5, the Green function is reduced to:


(2) $$G=\frac{3}{4\pi Er^{3}}\left(\begin{array}{cc} r^{2}+x^{2} & xy\\ xy & r^{2}+y^{2} \end{array}\right).$$

From these equations, the inversion of Equation (1) to calculate the stress field knowing the displacement field and the substrate modulus *E* is done through methods based on FTTC.

The main issue is that this inversion is an ill-posed problem which means that it can have several solutions which can be more or less realistic. Nevertheless, an ill-posed inverse problem can be solved by regularization which consists of introducing an additional parameter λ that stabilizes the solution. Consequently, the goal is to look for a force ***f*** minimizing:


(3) $$J={\left|Gf-u\right|}^{2}+\lambda^{2}{\left|f\right|}^{2},$$

where ***f*** and ***u*** are the force and the displacement field, respectively, ***G*** is the operator obtained from the Green function [Equations (1) and (2)]; λ governs the relative importance of experimental data [first term in Equation (3)] and the amplitude of the solution (second term) during minimization of ***J***.

The force reconstruction routine used in JEasyTFM is mainly based on previous work (Martiel *et al.* 2015) where the regularization factor has to be set by the user. Here, we determine the optimal regularization factor value by using a binary (or half-interval) search algorithm (Cormen *et al.* 2022).

To allow precise localization of the forces, the software generates super-imposed traction stress and cell images enabling mapping of the forces at a resolution up to 800 nm (Fig. 1d). The software allows also selection of cells of interest for automated tracking and segmentation. This allows the calculation of the total traction force value by integrating the selection obtained by segmentation of the cells over an extracted traction force modulus image. To normalize the total traction force with the cell area, the force ratio is also determined by dividing the total traction force value with the selection area. JEasyTFM automatically extracts all these values over the time series (Fig. 1e). This is fundamental in order to be able to correlate cell fate decision like adhesion, polarization, migration or division, and total forces (Fig. 1f, Supplementary Video S1). Additionally, as traction forces are generated by cellular actin-myosin system and transmitted to the extracellular matrix through focal adhesions, JEasyTFM provide also a focal adhesions segmentation module using a simple threshold process applied on fluorescently marked focal adhesion images. The sum of traction stresses per focal adhesion area can thus be calculated by integrating traction vectors over the region of interest.

The automated selection and alignment of the best focused images together with tracking and segmentation of the cells give this software a considerable advantage over other routines developed for TFM under ImageJ or Matlab, which are unable to analyze time-series experiments without a long and tedious process of image alignment and cropping. Nevertheless, within the up to date version, our package is not able to perform Monolayer Stress Microscopy analysis, a feature already available within other tools (Bauer *et al.* 2021). It should also be noted that JEasyTFM package is not adapted for the analysis of 3D TFM data where other algorithms have to be considered (Hazlett *et al.* 2020, Lekka *et al.* 2021, Delanoë-Ayari *et al.* 2023).

In summary, JEasyTFM, providing the acquisition is performed in the optimal conditions provides a complete solution for large data compilation, thus being suitable for force-based screening and video TFM analysis.

## Supplementary Material

vbad156_Supplementary_Data

## Data Availability

JEasyTFM together with a “Quick installation Evaluation &Testing” can be accessed through: http://questpharma.u-strasbg.fr/JEasyTFM.html. A complete user manual for the software can be found here: http://questpharma.u-strasbg.fr/JEasyTFM_manual.html. The original raw pictures used for the figures and demonstration can be downloaded from the following link: https://doi.org/10.57745/VAQNU8. The source code of the main software and all its dependencies can be found within the following software development and version control submitted to curation depositary https://hal.science/hal-03954798#.
